# Association of serum levels of fibrosis-related biomarkers with disease activity in patients with IgG4-related disease

**DOI:** 10.1186/s13075-018-1777-7

**Published:** 2018-12-14

**Authors:** Shin-ya Kawashiri, Tomoki Origuchi, Masataka Umeda, Ayako Nishino, Toshimasa Shimizu, Shoichi Fukui, Tomohiro Koga, Naoki Iwamoto, Kunihiro Ichinose, Mami Tamai, Hideki Nakamura, Takahiro Maeda, Mitsuhiro Kawano, Motohisa Yamamoto, Yasumori Izumi, Atsushi Kawakami

**Affiliations:** 1 Department of Community Medicine, Nagasaki University Graduate School of Biomedical Sciences, 1-12-4 Sakamoto, Nagasaki, 852-8523 Japan; 2 Immunology and Rheumatology, Nagasaki University Graduate School of Biomedical Sciences, Nagasaki, Japan; 3 Department of Rehabilitation Sciences, Nagasaki University Graduate School of Biomedical Sciences, Nagasaki, Japan; 4 Medical Education Development Center, Nagasaki University Hospital, Nagasaki, Japan; 5 Center for Comprehensive Community Care Education Medicine, Nagasaki University Graduate School of Biomedical Sciences, Nagasaki, Japan; 6 Clinical Research Center, Nagasaki University Hospital, Nagasaki, Japan; 7 Center for Bioinformatics and Molecular Medicine, Nagasaki University Graduate School of Biomedical Sciences, Nagasaki, Japan; 8 Division of Rheumatology, Kanazawa University Graduate School of Medicine, Kanazawa, Japan; 9 Department of Rheumatology, Sapporo Medical University School of Medicine, Sapporo, Japan; 10 Department of General Internal Medicine, NHO National Nagasaki Medical Center, Omura, Japan

**Keywords:** IgG4-related disease, Fibrosis, GDF-15 organ involvement, Prednisolone, Responder index

## Abstract

**Background:**

The aim of this study was to identify fibrosis-related serological surrogate outcome measures in patients with immunoglobulin G4-related disease (IgG4-RD).

**Methods:**

This was a clinical observational study of 72 patients with untreated IgG4-RD from four institutions in Japan. The serum concentrations of growth differentiation factor 15 (GDF-15), CCL2, hyaluronic acid (HA), amino-terminal propeptide of type III procollagen (PIIINP), and tissue inhibitor of metalloproteinases 1 (TIMP-1) were measured by enzyme-linked immunosorbent assays. The enhanced liver fibrosis (ELF) score was calculated from the TIMP-1, PIIINP, and HA values. We evaluated associations between the values of these biomarkers and laboratory data, the IgG4-RD responder index (IgG4-RD RI) score, and organ involvements.

**Results:**

Compared with the 44 healthy controls, the patients with IgG4-RD showed significantly elevated serum concentrations of GDF-15, MCP-1, HA, PIIINP, and TIMP-1 and ELF scores. The patients’ serum concentrations of GDF-15, CCL2, HA, and TIMP-1 (but not PIIINP) were positively correlated with each other. Among them, serum GDF-15 most efficiently distinguished patients with IgG4-RD from healthy controls. Serum GDF-15 was not associated with the IgG4-RD RI score or the number of organ involvements but was independently associated with the presence of retroperitoneal fibrosis and with parotid gland involvement.

**Conclusions:**

We observed increased serological surrogate outcome measures of fibrosis in IgG4-RD. GDF-15 may precisely reflect the fibrotic degree in patients with IgG4-RD.

**Electronic supplementary material:**

The online version of this article (10.1186/s13075-018-1777-7) contains supplementary material, which is available to authorized users.

## Background

Immunoglobulin G4-related disease (IgG4-RD) is a fibro-inflammatory condition generally characterized by tumefactive lesions, a dense lymphoplasmacytic infiltrate rich in IgG4-positive plasma cells, storiform fibrosis, and (often but not always) elevated serum IgG4 concentrations [1]. IgG4-RD, initially described in a cohort of Japanese patients with sclerosing pancreatitis [2, 3], has now been reported across an ethnically diverse spectrum in nearly every organ [1, 4–6]. IgG4-RD generally responds to glucocorticoids (GCs) in its inflammatory stage, but recurrent or refractory cases are common [4].

Fibrosis and the infiltration of IgG4-positive plasma cells are predominant histopathological features of IgG4-RD. Although histopathological findings are the key to the diagnosis of IgG4-RD, a tissue biopsy may not be anatomically available depending on the affected organ(s) (for example, the retroperitoneal space, bile duct, and pancreas). The extent of fibrosis can predict poor responsiveness to immunosuppressive therapies [4]. Therefore, fibrosis is an important element of pathogenesis in IgG4-RD. Transforming growth factor beta (TGFβ) and IL-10, which are Foxp3-positive regulatory T (Treg) cell cytokines associated with fibrosis, have been demonstrated to be overexpressed in the tissues of patients with IgG4-RD [7–10]. However, the serum concentrations of these cytokines were below detectable levels in patients with IgG4-RD [8]. Thus, serological surrogate cytokines and chemokines of fibrosis in IgG4-RD have not yet been revealed. In this regard, Akiyama et al. recently showed that CC chemokine ligand 18 (CCL18) is a useful biomarker associated with disease activity in IgG4-RD [11].

The enhanced liver fibrosis (ELF) score is both a clinically validated surrogate score of the severity of tissue fibrosis and a predictor of clinical outcomes in the setting of chronic fibrotic liver diseases [12]. The ELF score assesses the degree of extracellular matrix deposition by measuring the serum concentrations of three analytes in both fibrogenesis and the remodeling of the extracellular matrix: hyaluronic acid (HA), amino-terminal propeptide of type III procollagen (PIIINP), and tissue inhibitor of metalloproteinases 1 (TIMP-1) [12, 13]. It has also been shown that the ELF score is correlated with fibrosis in IgG4-RD [13] and systemic sclerosis (SSc) [14].

In addition to CCL18 and the ELF score, growth differentiation factor 15 (GDF-15) and CCL2 are thought to be involved in the fibrotic manifestation of human diseases such as SSc [15–18]. GDF-15, also known as macrophage inhibitory cytokine-1 (MIC-1), is a member of the TGFβ superfamily. GDF-15 was originally identified as a factor secreted by activated macrophages [19]. Serum levels of GDF-15 are elevated in patients with SSc, and GDF-15 appears to reflect a general role in the fibrotic process [15]. The chemokine CCL2, also known as monocyte chemotactic protein-1 (MCP-1), is considered an important chemotactic mediator of macrophages. Similarly, elevated serum CCL2 is predominantly associated with fibrotic manifestations in patients with SSc [17, 18]. It was also demonstrated that GDF-15 acts as a macrophage recruitment signal through CCL2 and its receptor CCR2 [20]. We conducted the present study to explore the roles of circulating biomarkers, especially GDF-15, that may reflect the fibrotic degree in patients with IgG4-RD.

## Materials and methods

### Patients

We enrolled the 72 consecutive untreated patients who fulfilled the comprehensive diagnostic criteria for IgG4-RD [21] or organ-specific criteria [22–28] and received treatment at one of the following four institutions in Japan during the period from January 2010 to January 2016: Nagasaki University Hospital, Kanazawa University Hospital, Sapporo Medical University Hospital, and the NHO National Nagasaki Medical Center. The patients’ clinical data pertaining to demographics, laboratory findings, and treatment were obtained from their medical records. Disease durations were calculated from the time point at which the patient first noticed the symptoms ultimately attributed to IgG4-RD or the time point at which the disease was first recognized. Organ involvement was determined by a review of the patient’s history, physical examination findings, imaging results, laboratory studies, and tissue biopsies. Imaging included ultrasound, computed radiography, computed tomography (CT), magnetic resonance imaging, and positron emission tomography/CT.

No patients with hepatic involvement by IgG4-RD or chronic fibrotic liver disease were enrolled. Sixty patients underwent a biopsy, and the diagnoses of 51 patients were confirmed by histopathology (IgG4-positive cells greater than 10 high-power fields, IgG4-positive cells/IgG-positive cells ratio greater than 40%). In 12 other patients, a tissue biopsy was not anatomically available. None of the patients was on GC treatment at baseline. Thirteen patients (from Nagasaki University Hospital) were also evaluated after GC treatment.

This study was a retrospective observational study using anonymized information. The patients gave their informed consent to be subjected to the protocol, which was approved by the institutional review board of each institution.

### Biomarker measurement

Baseline serum samples were collected from untreated 72 patients at the time of diagnosis. Serum samples at sustained remission after induction of GC treatment were collected from 13 patients. Serum samples were aliquoted and stored at − 20 °C until analysis. The serum levels of GDF-15 (R&D Systems, Minneapolis, MN, USA), CCL2 (MCP-1) (R&D Systems), TIMP-1 (R&D Systems), and HA (R&D Systems) and PIIINP (USCN Life, Wuhan, China) were measured with specific enzyme-linked immunosorbent assay kits. The assays were calibrated using standards provided by the manufacturers. These biomarkers were also measured in 44 healthy controls (median age was 35 years and 32% were male). The ELF score provides a single value obtained with an algorithm combining the quantitative serum measurements of TIMP-1, PIIINP, and HA [12, 29]. The ELF score was calculated directly using the following formula:

$$ \mathrm{ELF}\ \mathrm{score}=2.494+0.846\ \ln\ \left({\mathrm{C}}_{\mathrm{HA}}\right)+0.735\ \ln\ \left({\mathrm{C}}_{\mathrm{PIIINP}}\right)+0.391\ \ln\ \left({\mathrm{C}}_{\mathrm{TIMP}-1}\right). $$

### Statistical analyses

Statistical analysis was performed by using JMP Pro statistical software, version 11.0 (SAS Institute, Cary, NC, USA). Quantitative variables are presented as medians and interquartile ranges (IQRs). Categorical variables are presented as percentages. We used the Mann–Whitney *U* test for comparisons between independent medians, and we used the chi-squared test for the evaluation of the associations between categorical variables. Correlations were assessed with Spearman’s correlation coefficient. The changes in the serum GDF-15 concentration from baseline were analyzed by using the Wilcoxon signed-rank test. We investigated the organ involvements associated with high GDF-15 (that is, higher than the median at baseline: GDF-15 of at least 1121 pg/mL) by performing a multivariate logistic regression analysis. Variables with *P* values of less than 0.05 in a univariate model were used in the multivariate model. The overall significance level for statistical analysis was 5% (two-sided). *P* values of less than 0.05 were accepted as significant.

## Results

### Patient characteristics

The demographic and clinical characteristics of the 72 patients with IgG4-RD are summarized in Table 1. The involved organs (in descending order) were the submandibular glands (*n* = 49 cases, 68.1%), lymph nodes (n = 49, 68.1%), lacrimal glands (*n* = 38, 52.8%), pancreas (*n* = 23, 31.9%), retroperitoneal fibrosis (*n* = 21, 29.2%), lungs (*n* = 16, 22.2%), parotid glands (*n* = 13, 18.2%), kidneys (*n* = 12, 16.7%), orbits (*n* = 10, 13.9%), prostate (*n* = 7, 9.7%), bile duct (*n* = 6, 8.3%), thyroid (n = 4, 5.6%), aorta (n = 2, 2.8%), and pituitary gland (n = 1, 1.4%). A median of three (range of one to eight) organs per patient were involved. Fifty-two (72.2%) patients had at least three organs involved.

**Table 1:** Demographic, clinical, and laboratory characteristics of the 72 patients with immunoglobulin G4-related disease

	IgG4-RD (*n* = 72)
Age, years	66 (61–72)
Male	45 (62.5)
Disease duration, months	11 (5–34)
Initial treatment	Glucocorticoids; 57 (79.2)
	Observation; 15 (20.8)
Laboratory data	
Total-IgG, mg/dL	1900 (1490–2480)
IgG4, mg/dL	457 (260–705)
IgG4 ≥135 mg/dL	68/72 (94.4)
IgG4/total IgG, %	23.9 (15.4–34.3)
IgE, U/mL	352 (130–681)
CH50, U/mL	43.6 (37.5–53.8)
C3, mg/dL	85.5 (75.0–99.9)
C4, mg/dL	19.7 (13.0–27.1)
sIL-2R, U/mL	762 (467–1093)

The data are median (interquartile range, Q_1–4_–Q_3/4_) or number (percentage). Abbreviations: *IgG4-RD* immunoglobulin G4-related disease, *sIL-2R* soluble interleukin-2 receptor

The median (IQR) of serum IgG4 concentrations was 457 (260–705) mg/dL, and the concentration was at least 135 mg/dL in 68 patients (94.4%). Regarding serum complement levels, low C3 (<65 mg/dL), low C4 (<13 mg/dL), and low CH50 (<30 U/mL) concentrations were seen in 15.2%, 24.6%, and 19.4% of the patients, respectively. Two or more serum complements were depressed in 13 patients (18.1%), and kidney was an involved organ in seven of these patients (53.8%). The serum concentration of soluble interleukin-2 receptor (sIL-2R) (normal range 127–582 U/mL) was elevated in 34 of the 58 patients tested (58.6%), and the serum concentration of IgE (normal range <269 U/mL) was elevated in 35 of the 60 patients tested (58.3%). The median (IQR) of IgG4-RD responder index (IgG4-RD RI) score at baseline, calculated according to Carruthers et al. [30], was 9 (6–12).

Fifty-seven patients (79.2%) initially received treatment with GCs depending on the treatment guidelines for each organ involvement, and no patients were treated with any other therapies. The median initial dose of GC was 30 mg daily. Other patients were followed without any treatment.

### Comparison of biomarkers between the patients with IgG4-RD and healthy controls

**Fig. 1:**
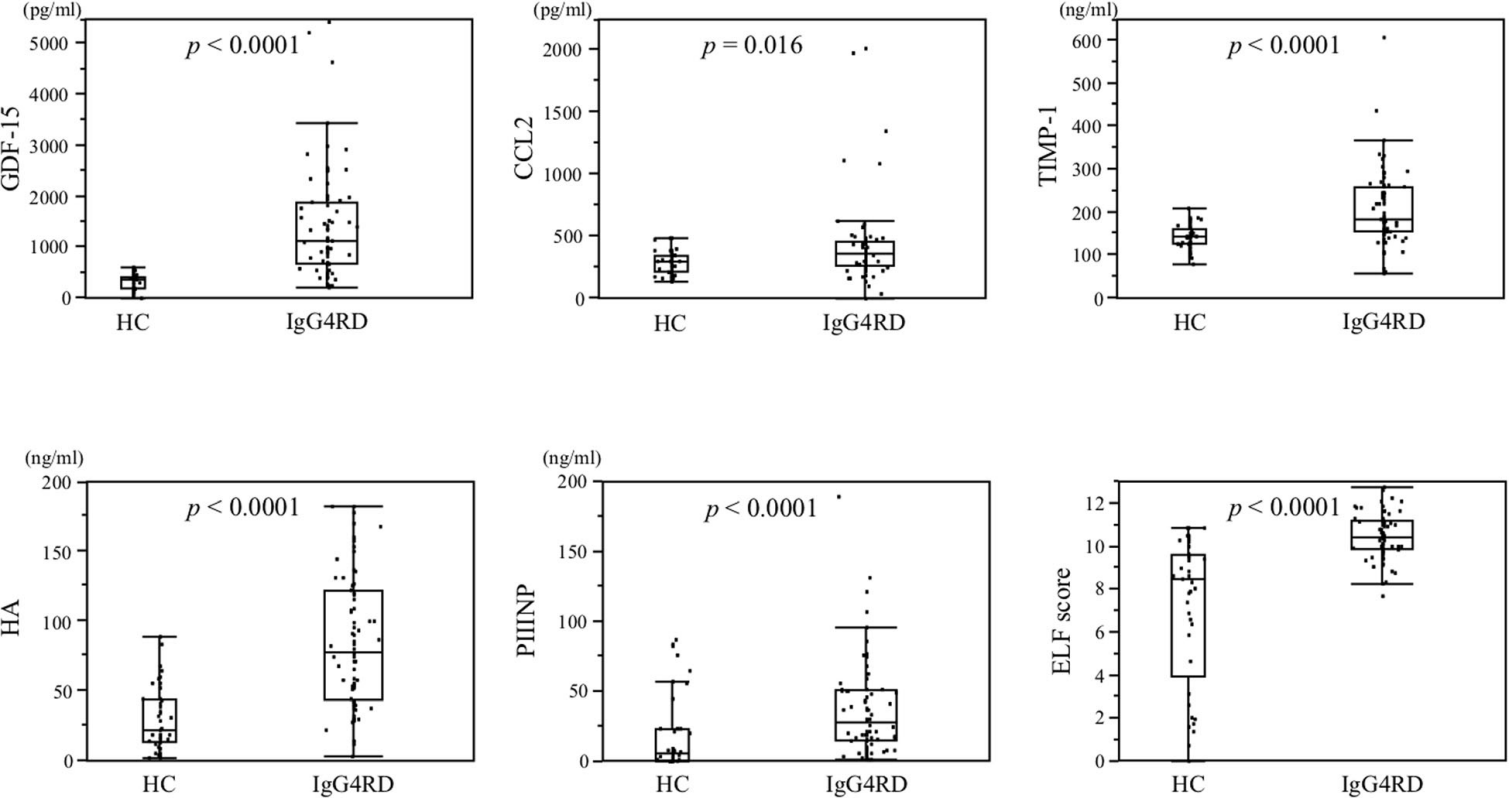
Comparison of biomarkers between patients with IgG4-RD and healthy controls. The serum concentrations of GDF-15, CCL2, HA, PIIINP, and TIMP-1 were elevated significantly among the patients with IgG4-RD compared with the healthy controls. Within-group comparisons were made by using Mann–Whitney *U* test. Line chart, mean; horizontal bar, median; boxes, 25th and 75th percentiles; bars, 5th and 95th percentiles. Abbreviations: *CCL2* CC chemokine ligand 2, *ELF* enhanced liver fibrosis, *GDF-15* growth differentiation factor 15, *HA* hyaluronic acid, *IgG4-RD* immunoglobulin G4-related disease, *PIIINP* amino-terminal propeptide of type III procollagen, *TIMP-1* tissue inhibitor of metalloproteinases 1

The serum concentrations of GDF-15, CCL2, TIMP-1, HA, and PIIINP were significantly higher in the patients with IgG4-RD compared with the healthy controls (Table 2 and Fig. 1). The ELF score was also significantly higher in the patients with IgG4-RD compared with the healthy controls (*P* <0.0001, Table 2 and Fig. 1), although the age and gender distribution were statistically different between patients with IgG4-RD and healthy controls (younger and low proportion of males in healthy controls compared with patients with IgG4-RD , respectively). The cutoff values for distinguishing the patients with IgG4-RD from the healthy controls were as follows: GDF-15, 666 pg/mL (area under the curve 0.92, sensitivity 77.8%, specificity 100%); CCL2, 389 pg/mL (0.68, 39.7%, 90.2%); TIMP-1, 154 ng/mL (0.78, 77.8%, 69.8%); HA, 65.1 ng/mL (0.85, 61.1%, 93%); PIIINP, 9.0 ng/mL (0.74, 81.9%, 68.3%), and the ELF score, 9.7 (0.85, 79.2%, 78.1%). The serum concentrations of these biomarkers were not correlated with disease durations in the patients with IgG4-RD (data not shown).

**Table 2:** Comparison of biomarkers between patients with immunoglobulin G4-related disease and healthy controls

	IgG4-RD *n* = 72	Healthy controls *n* = 44	*P* value
GDF-15, pg/mL	1121 (680–1839)	362 (191–433)	<0.0001
CCL2, pg/mL	357 (268–449)	300 (219–349)	0.016
TIMP-1, ng/mL	183 (156–260)	143 (127–163)	<0.0001
HA, ng/mL	78.1 (43.6–121)	22.6 (13.9–43.6)	<0.0001
PIIINP, ng/mL	27.2 (14.5–50.4)	5.8 (0.4–23.0)	<0.0001
ELF score	10.4 (9.8–11.2)	8.4 (4.6–9.5)	<0.0001

The data are median (interquartile range, Q_1/4_–Q_3/4_). Within-group comparisons were assessed with the Mann–Whitney *U* test. Abbreviations: *CCL2* CC chemokine ligand 2, *ELF* enhanced liver fibrosis, *GDF-15* growth differentiation factor 15, *HA* hyaluronic acid, *IgG4-RD* immunoglobulin G4-related disease, *PIIINP* amino-terminal propeptide of type III procollagen, *TIMP-1* tissue inhibitor of metalloproteinases 1

### Correlations among the biomarkers

The patients’ serum concentrations of GDF-15, CCL2, HA, and TIMP-1 (but not PIIINP) were positively correlated with each other (Table 3). The ELF scores were positively correlated with the serum levels of GDF-15 and CCL2. The serum levels of total-IgG, IgG4, and sIL-2R were positively correlated with each other and were negatively correlated with the levels of the serum complements C3, C4, and CH50. In addition, the serum levels of GDF-15 and TIMP-1 were negatively correlated with or tended to be negatively correlated with those of the complements. The serum levels of GDF-15, CCL2, and TIMP-1 were positively correlated with the sIL-2R level.

**Table 3:** Correlations between each biomarker in patients with immunoglobulin G4-related disease

	GDF-15		CCL2		TIMP-1		HA		PIIINP		ELF score		Total-IgG		IgG4		CH50		C3		C4		sIL2R	
	*rs*	*P* value	*rs*	*P* value	*rs*	*P* value	*rs*	*P* value	*rs*	*P* value	*rs*	*P* value	*rs*	*P* value	*rs*	*P* value	*rs*	*P* value	*rs*	*P* value	*rs*	*P* value	*rs*	*P* value
GDF-15	–	–	0.48	<0.0001	0.62	<0.0001	0.61	<0.0001	0.13	0.27	0.51	<0.0001	0.37	0.0013	0.12	0.33	−0.24	0.061	−0.38	0.0016	−0.34	0.0049	0.47	0.0002
CCL2	0.48	<0.0001	–	–	0.50	<0.0001	0.47	<0.0001	−0.031	0.80	0.35	0.0032	0.13	0.10	0.12	0.33	−0.14	0.31	−0.25	0.0085	−0.22	0.085	0.35	0.0064
TIMP-1	0.62	<0.0001	0.50	<0.0001	–	–	0.29	0.015	0.06	0.64	0.36	0.0021	0.25	0.036	0.10	0.43	−0.35	0.005	−0.21	0.095	−0.21	0.095	0.43	0.0007
HA	0.61	<0.0001	0.47	<0.0001	0.29	0.015	–	–	0.06	0.64	0.65	<0.0001	0.30	0.011	0.18	0.14	−0.18	0.17	−0.23	0.069	−0.24	0.055	0.26	0.051
PIIINP	0.13	0.27	−0.31	0.80	0.06	0.64	0.06	0.64	–	–	0.72	<0.0001	0.02	0.89	−0.02	0.87	−0.12	0.34	−0.18	0.16	−0.12	0.33	0.04	0.74
ELF score	0.51	<0.0001	0.35	0.0032	0.36	0.0021	0.65	<0.0001	0.72	<0.0001	–	–	0.24	0.044	0.09	0.44	−0.10	0.43	−0.21	0.084	−0.18	0.16	0.22	0.098
Total-IgG	0.37	0.0013	0.13	0.28	0.25	0.036	0.30	0.011	0.02	0.89	0.24	0.044	–	–	0.74	<0.0001	−0.34	0.0067	−0.43	0.0003	−0.40	0.001	0.69	<0.0001
IgG4	0.12	0.33	0.12	0.33	0.10	0.43	0.18	0.14	−0.02	0.87	0.09	0.44	0.74	<0.0001	–	–	−0.29	0.023	−0.40	0.0009	−0.37	0.0025	0.60	<0.0001
CH50	−0.24	0.061	−0.14	0.31	−0.35	0.005	−0.18	0.17	−0.12	0.34	−0.10	0.43	−0.34	0.0067	−0.29	0.023	–	–	0.56	<0.0001	0.66	<0.0001	−0.50	0.0003
C3	−0.38	0.0016	−0.25	0.051	−0.21	0.084	−0.23	0.069	−0.18	0.16	−0.21	0.084	−0.43	0.0003	−0.4	0.0009	0.56	<0.0001	–	–	0.73	<0.0001	−0.54	0.0002
C4	−0.34	0.0049	−0.22	0.085	−0.21	0.095	−0.24	0.055	−0.12	0.33	−0.18	0.16	−0.40	0.001	−0.37	0.0025	0.66	<0.0001	0.73	<0.0001	–	–	−0.39	0.0038
sIL-2R	0.47	0.0002	0.35	0.0064	0.43	0.0007	0.26	0.051	0.04	0.74	0.22	0.098	0.69	<0.0001	0.60	<0.0001	−0.50	0.0003	−0.54	0.0002	−0.39	0.0038	–	–

The results were obtained using Spearman’s correlation coefficient. Abbreviations: *CCL2* CC chemokine ligand 2, *ELF* enhanced liver fibrosis, *GDF-15* growth differentiation factor 15, *HA* hyaluronic acid, *IgG4-RD* immunoglobulin G4-related disease, *PIIINP* amino-terminal propeptide of type III procollagen, *sIL-2R* soluble interleukin-2 receptor, *TIMP-1* tissue inhibitor of metalloproteinases 1

### Correlations between organ involvement and biomarkers

The patients’ serum concentrations of GDF-15, CCL2, HA, TIMP-1, and PIIINP and the ELF score were not correlated with the IgG4-RD RI score or the number of organ involvements (Table 4). In contrast, the serum levels of total-IgG, IgG4, and sIL-2R were positively correlated with the IgG4-RD RI score or the number of organ involvements or both. The serum levels of C3 and C4 were negatively correlated with and that of CH50 tended to be negatively correlated with the IgG4-RD RI score or the number of organ involvements or both.

**Table 4:** Correlations between the immunoglobulin G4-related disease responder index score or the number of organ involvements and the biomarkers

	IgG4-RD responder index		Number of organ involvements	
	*rs*	*P* value	*rs*	*P* value
GDF-15	0.11	0.35	0.07	0.54
CCL2	0.07	0.56	0.03	0.81
TIMP-1	0.11	0.36	0.10	0.42
HA	0.17	0.16	0.17	0.15
PIIINP	−0.19	0.10	−0.17	0.16
ELF score	−0.07	0.58	−0.04	0.73
IgG	0.49	<0.0001	0.43	0.0002
IgG4	0.64	<0.0001	0.61	<0.0001
CH50	−0.21	0.10	−0.24	0.063
C3	−0.27	0.029	−0.28	0.022
C4	−0.33	0.0066	−0.35	0.0043
sIL-2R	0.52	<0.0001	0.51	<0.0001

The results were obtained using Spearman’s correlation coefficient. *Abbreviations: CCL2* CC chemokine ligand 2, *ELF* enhanced liver fibrosis, *GDF-15* growth differentiation factor 15, *HA* hyaluronic acid, *IgG4-RD* immunoglobulin G4-related disease, *PIIINP* amino-terminal propeptide of type III procollagen, *sIL-2R* soluble interleukin-2 receptor, *TIMP-1* tissue inhibitor of metalloproteinases 1

### Association of serum GDF-15 with involved organs and changes in the serum GDF-15 level after GC treatment

We attempted to identify the organ involvements associated with a high GDF-15 level (that is, higher than the median at baseline: GDF-15 of at least 1121 pg/mL) by performing a multivariate logistic regression analysis (Additional file 1: Table S1). The presence of retroperitoneal fibrosis (odds ratio (OR) 3.47, 95% confidence interval (CI) 1.2–11.4, *P* = 0.026) and the presence of parotid gland involvement (OR 3.92, 95% CI 1.0–19.5, *P* = 0.048) were independently associated with high GDF-15. Since GDF-15 was high in male patients compared with female patients (*P* = 0.0008) and the positive correlation of GDF-15 with age was present (rs = 0.53, *P* <0.0001), a multivariate logistic regression analysis was adjusted by age and gender which still showed the tendency (parotid glands OR 4.10, 95% CI 0.99–21.8, *P* = 0.053; retroperitoneal fibrosis OR 2.53, 95% CI 0.77–8.87, *P* = 0.13).

**Fig. 2:**
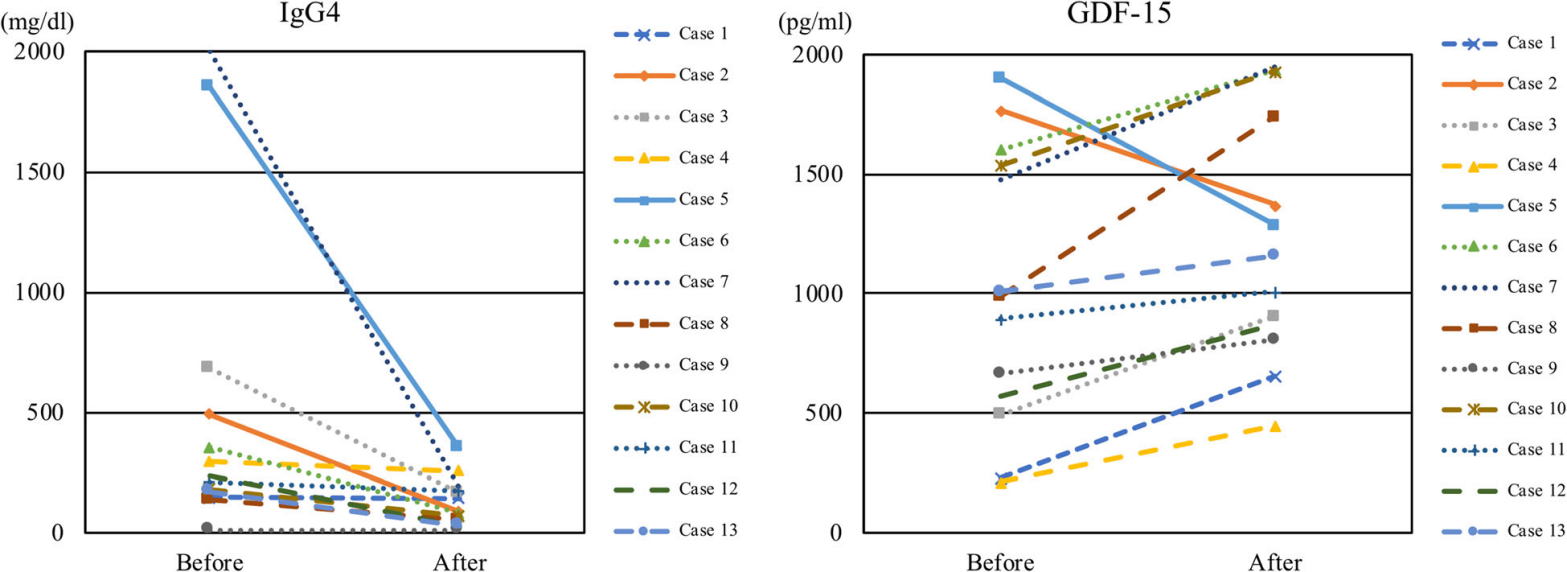
Changes of serum immunoglobulin G4 (IgG4) and GDF-15 concentrations after glucocorticoid treatment in 13 patients with IgG4-related disease. Abbreviation: *GDF-15* growth differentiation factor 15

The changes in the levels of serum biomarkers after induction of GC treatment in 13 patients are summarized in Additional file 1: Table S2 and Fig. 2. The median GC treatment period was 3.5 years. After the patients’ GC treatment, their median (range) dose of prednisolone was reduced from 35 (25–40) mg to 3 (1–5) mg daily. Radiographic improvement of organ involvements was confirmed in all 13 patients. The serum concentration of IgG4 was decreased in all patients (median 236–88 mg/dL, *P* = 0.0005). The serum concentrations of GDF-15, CCL2, and TIMP-1 were increased. The serum concentrations of HA and PIIINP as well as the ELF score did not change during GC treatment.

## Discussion

In regard to the serum cytokines and chemokines of patients with IgG4-RD, the associations of CCL18 [11] and the ELF score [13] with the disease activity of IgG4-RD have been shown. As reported in the representative fibrotic disease SSc, Akiyama et al. demonstrated that the serum CCL18 level was positively correlated with the IgG4-RD RI score, the number of affected organs, and the serum levels of IgG4 and sIL-2R [11]. The ELF score was found to be a clinically useful indicator of active fibrosis and the extent of IgG4-RD [13]. These data indicate the importance of serum biomarkers reflecting the fibrotic process in patients with IgG4-RD and therefore we focused on the significance of GDF-15 and CCL2 in our present investigation.

As reported previously, we observed that the ELF score was increased in patients with IgG4-RD. We also observed that the serum concentrations of GDF-15 and CCL2 were significantly high in the patients compared with the healthy controls. The serum concentration of GDF-15 was the most useful for distinguishing patients with IgG4-RD from healthy controls. This is the first report of associations of GDF-15 and CCL2 with IgG4-RD. There is a close relation between GDF-15 and CCR2. GDF-15 upregulates CCL2 and its receptor CCR2 expression in macrophages and promotes macrophage chemotaxis [16, 19], suggesting that GDF-15 production from infiltrated macrophages may enhance further macrophage recruitment by augmenting the expressions of CCL2 and CCR2, leading to the development of fibrosis.

A recent study demonstrated that the CCL2–CCR2 axis regulates the macrophage polarization of M2 dominant by influencing the expression of functionally relevant and polarization-associated genes and down-modulating pro-inflammatory cytokine production [31]. Furukawa et al. suggested that M2 macrophages are involved in the process of fibrosis via the recruitment of circulating fibroblast precursors (fibrocytes) [32]. They showed that preferential M2 macrophages contribute to fibrosis in the submandibular glands of patients with IgG4-related dacryoadenitis and sialoadenitis (IgG4-DS) [32]. Therefore, GDF-15 may contribute to the fibrotic process of IgG4-RD by forming and activating M2 macrophages through the CCL2–CCR2 axis.

Our present findings revealed the association of both GDF-15 and CCR2 with IgG4-RD, but there are remaining clinical outcomes that could not be explained by GDF-15 or CCR2. First, although the serum concentration of GDF-15 was correlated with some organ involvements of IgG4-RD, we observed that the conventional serum markers, including IgG4, complements, and sIL-2R, were correlated with the IgG4-RD RI score or the number of organ involvements better than GDF-15 or CCL2. Second, a clinical response to prednisolone was shown by our data, but the serum concentrations of GDF-15 and CCL2 were even elevated in the prednisolone-treated patients. It was reported that B-cell depletion therapy with rituximab reduced the IgG4-RD RI score, ELF score, and myofibroblast activation [13]. The prescription of longitudinal GCs was not reported in that study [13], unlike the present study. The ELF scores of the present patients who received prednisolone were not decreased. Akiyama et al. found a significant reduction of the serum CCL18 concentration along with a reduction of the IgG4-RD RI score by prednisolone therapy but did not examine the ELF score. Taken together, the previous and present findings lead us to refer to the limitations of GDF-15 and CCL2 reflecting disease activity and the fibrotic process of IgG4-RD.

In addition to serum biomarkers, changes in the numbers of circulating plasmablasts [33, 34], activated follicular helper 2 T cells [33–35], activated follicular helper 1 T cells [34, 35], and CD4^+^ cytotoxic T cells [36] were observed in patients with IgG4-RD. The numbers of these cell subsets were decreased by GCs or rituximab. A combined examination of serum biomarkers, including GDF-15 and CCL2, the ELF score, and the immunophenotyping of lymphocytes is thus needed for a further understanding of our present findings.

## Conclusions

We observed increased serological surrogate outcome measures of fibrosis in patients with IgG4-RD. GDF-15 may precisely reflect the fibrotic degree in patients with IgG4-RD.

## Additional file

Additional file 1: **Table S1.** Association between growth differentiation factor 15 (GDF-15) and organ involvements. **Table S2.** Changes of serum biomarkers and immunoglobulin G4-related disease responder index (IgG4-RD RI) score after glucocorticoid treatment in 13 patients. (DOCX 18 kb)

## Abbreviations

CCL: CC chemokine ligand
CI: Confidence interval
CT: Computed tomography
ELF: Enhanced liver fibrosis
GC: Glucocorticoid
GDF-15: Growth differentiation factor 15
HA: Hyaluronic acid
IgG4-RD: Immunoglobulin G4-related disease
IQR: Interquartile range
MCP-1: Monocyte chemotactic protein-1
PIIINP: Amino-terminal propeptide of type III procollagen
sIL-2R: Soluble interleukin-2 receptor
SSc: Systemic sclerosis
TGFβ: Transforming growth factor beta
TIMP-1: Tissue inhibitor of metalloproteinases 1
