# Supplementary material for: Association of serum levels of fibrosis-related biomarkers with disease activity in patients with IgG4-related disease
Source: Arthritis Res Ther. 2018 Dec 14;20:277. doi: 10.1186/s13075-018-1777-7 (PMC6293522; doi:10.1186/s13075-018-1777-7)
Supplement: Supplementary file 1 — Table S1. Association between growth differentiation factor 15 (GDF-15) and organ involvements. Table S2. Changes of serum biomarkers and immunoglobulin G4-related disease responder index (IgG4-RD RI) score after glucocorticoid treatment in 13 patients. (DOCX 18 kb) [file 13075_2018_1777_MOESM1_ESM.docx]

**Table S1.** Association between GDF-15 and organ involvements

|  | **GDF-15** | | | | | | |
| --- | --- | --- | --- | --- | --- | --- | --- |
|  | **Univariate model** | | | **Multivariate model** | | | |
|  | **Low** | **High** | ***p*** | | **OR** | **95% CI** | ***p*** |
| Pituitary gland | 0 (0) | 1 (100) | 0.49 | |  |  |  |
| Orbit | 4 (40.0) | 6 (60.0) | 0.51 | |  |  |  |
| Lacrimal glands | 22 (57.9) | 16 (42.1) | 0.35 | |  |  |  |
| Parotid glands | 3 (23.1) | 10 (76.9) | 0.033 | | 3.92 | 1.01–19.5 | 0.048 |
| Submandibular glands | 26 (53.1) | 23 (46.9) | 0.80 | |  |  |  |
| Thyroid | 2 (50.0) | 2 (50.0) | 1.00 | |  |  |  |
| Lung | 8 (50.0) | 8 (50.0) | 1.00 | |  |  |  |
| Pancreas | 15 (65.2) | 8 (34.8) | 0.13 | |  |  |  |
| Bile duct | 3 (50.0) | 3 (50.0) | 1.00 | |  |  |  |
| Kidney | 5 (41.7) | 7 (58.3) | 0.54 | |  |  |  |
| Retroperitoneal fibrosis | 6 (28.6) | 15 (71.4) | 0.019 | | 3.47 | 1.16–11.4 | 0.026 |
| Aorta | 1 (50.0) | 1 (50.0) | 1.00 | |  |  |  |
| Prostate | 2 (28.6) | 5 (71.4) | 0.25 | |  |  |  |
| Lymph nodes | 25 (51.0) | 24 (49.0) | 1.00 | |  |  |  |
| The data are number (percentage). Low, GDF-15 <1121 pg/ml. High, GDF-15 ≥1121 pg/ml. | | | | | | | |

**Table S2.** Changes of serum biomarkers and IgG4-RD RI score after glucocorticoid treatment in 13 patients

|  | **Before** | **After** | ***p*** |
| --- | --- | --- | --- |
| IgG4, mg/dl | 236 (170–490) | 88 (54–174) | 0.0005 |
| GDF-15, pg/ml | 989 (571–1537) | 1158 (872–1743) | 0.034 |
| CCL2, pg/ml | 310 (248–392) | 359 (297–434) | 0.033 |
| TIMP-1, ng/ml | 220 (172–245) | 246 (207–280) | 0.019 |
| HA, ng/ml | 70.6 (51.4–86.6) | 70.8 (55.4–91.0) | 0.97 |
| PIIINP, ng/ml | 20.4 (5.7–76.8) | 35.6 (28.2–57.4) | 0.86 |
| ELF score | 10.3 (9.4–10.9) | 10.8 (10.5–11.4) | 0.25 |
| IgG4-RD RI score | 6 (3-12) | 1 (1-2) | 0.0002 |
| The data are median (interquartile range, Q1–4–Q3/4). Within-group comparisons were assessed with the Wilcoxon signed ranks test. ELF, enhanced liver fibrosis; GDF-15, growth differentiation factor 15; HA, hyaluronic acid; IgG4-RD, IgG4-related disease; PIIINP, amino-terminal propeptide of type III procollagen; RI, responder index; TIMP-1, tissue inhibitor of metalloproteinases 1. | | | |
